# A new species of *Tenuibaetis* Kang & Yang, 1994 from Indonesia (Ephemeroptera, Baetidae)

**DOI:** 10.3897/zookeys.820.31487

**Published:** 2019-01-28

**Authors:** Thomas Kaltenbach, Jean-Luc Gattolliat

**Affiliations:** 1 Museum of Zoology, Palais de Rumine, Place Riponne 6, CH-1005 Lausanne, Switzerland Museum of Zoology Lausanne Switzerland; 2 University of Lausanne (UNIL), Department of Ecology and Evolution, CH-1015 Lausanne, Switzerland University of Lausanne Lausanne Switzerland

**Keywords:** Java, mayflies, morphology, new species, systematics

## Abstract

A new species of *Tenuibaetis* Kang & Yang is described and illustrated based on larvae collected in Java in 2010, which is the most southeastern report of this genus to date. The total number of *Tenuibaetis* species is thereby augmented to seven. The morphological differences of all other species are discussed and summarised in tabular form.

## Introduction

The family Baetidae has the highest species diversity among mayflies, comprising of more than 1000 species in 104 genera, which is approximately one quarter of all mayfly species known worldwide (Sartori and Brittain 2015). It has a cosmopolitan distribution with the exception of Antarctica and New Zealand. Investigations of the molecular phylogeny of the order Ephemeroptera revealed the relatively primitive status of the family (Ogden and Whiting 2005, Ogden et al. 2009).

The genus *Tenuibaetis* Kang & Yang (Kang et al. 1994) is a small genus of Baetidae, which includes six species: *T.flexifemora* (Gose), *T.pseudofrequentus* (Müller-Liebenau), *T.frequentus* (Müller-Liebenau & Hubbard), *T.arduus* (Kang & Yang), *T.inornatus* (Kang & Yang), and *T.parvipterus* Fujitani (Gose 1980, Müller-Liebenau 1985, Müller-Liebenau and Hubbard 1985, Kang et al. 1994, Fujitani et al. 2003a, 2011, Barber-James et al. 2013, Kubendran et al. 2015). Additionally, there are two species considered to belong to *Tenuibaetis* by Kluge (2018), *Baetisursinus* Kazlauskas, 1963 and *B.hissaricus* Novikova, 1991, but they were never formally assigned to this genus. The latter was originally described as subspecies of *B.ursinus*.

The distribution of *Tenuibaetis* is limited to Japan (*T.flexifemora*, *T.pseudofrequentus*, *T.parvipterus*; Gose 1980, Fujitani et al. 2003a, b, 2011, Fujitani 2008), Taiwan (*T.pseudofrequentus*, *T.arduus*, *T.inornatus*; Müller-Liebenau 1985, Kang et al. 1994), Hong-Kong (*T.pseudofrequentus*; Tong and Dudgeon 2000), India (*T.frequentus*; Balaji et al. 1990, Sivaramakrishnan and Venkataraman 1990, Kubendran et al. 2015), and Sri Lanka (*T.frequentus*; Müller-Liebenau and Hubbard 1985, Kubendran et al. 2015). *Baetishissaricus* was described from Tadjikistan (Novikova 1991) and *B.ursinus* is distributed in the Middle East, the Russian Far East, Mongolia, and Korea; details are given by Kluge (2018).

*Tenuibaetis* was originally considered as a subgenus of *Baetis* Leach with the type species Baetis (Tenuibaetis) pseudofrequentus (Kang et al. 1994). The subgeneric diagnosis was based on the following combination of larval characters: mandibles with smooth margin between prostheca and mola, without setae; the shape of the labial palpus (segment II poorly expanded at the inner distal margin, segment III conical); a well-developed femoral patch; and a patch of notched scales on the paraproct. Waltz and McCafferty (1997) assigned Baetis (Tenuibaetis) pseudofrequentus to the genus *Baetiella* Uéno, 1931 based on the shape of the labial palp and synonymised *Tenuibaetis* with *Baetiella*. Fujitani et al. (2003a, 2011) contradicted that opinion, mainly as they considered that species of *Tenuibaetis* can be separated from *Baetiella* by the inner margins of cerci fringed with setae in *Tenuibaetis* and glabrous in *Baetiella* and the presence of robust setae with median ridge on the anterior surface of the larval femur as the exclusive diagnostic character of *Tenuibaetis*. Consequently, they revalidated *Tenuibaetis*, removed *T.pseudofrequentus* from *Baetiella* and elevated *Tenuibaetis* to generic level (Fujitani et al. 2003a).

Here, a new species of *Tenuibaetis* is described, based on larvae collected in 2010 on the island of Java (Indonesia).

## Materials and methods

The specimens were preserved in 80% ethanol. The dissection of larvae was done in Cellosolve (2-Ethoxyethanol) under Olympus SZX7 stereomicroscope and mounted on slides with Euparal liquid.

Drawings were made using an Olympus BX43 microscope. Photographs of larvae were taken with a Canon EOS 6D camera and the Visionary Digital Passport imaging system (http://www.duninc.com) and processed with the programs Adobe Photoshop Lightroom (http://www.adobe.com) and Helicon Focus version 5.3 (http://www.heliconsoft.com). Photographs were subsequently enhanced with Adobe Photoshop Elements 13.

For the morphological terminology, we are referring to Hubbard (1995) and Morihara and McCafferty (1979).

## Taxonomic part

### Tenuibaetis fujitanii sp. n.

Taxon classificationAnimaliaEphemeropteraBaetidae

http://zoobank.org/FE2B0235-8030-49C2-9793-101C155846B4

Figures 1 , 2 , 3

#### Diagnosis.

**Figure 1.**
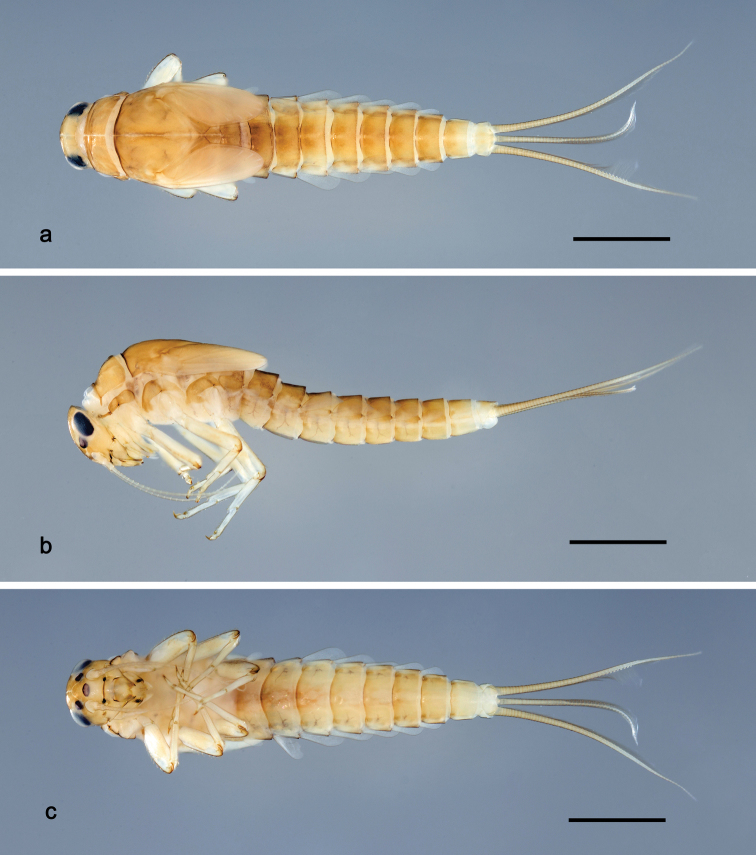
*Tenuibaetisfujitanii* sp. n., larva habitus: **a** Dorsal view **b** Lateral view **c** Ventral view. Scale bars: 1 mm.

***Larva.*** Following combination of characters: A) head, thorax and abdomen dorsally brown with some darker areas as in Fig. 1a; B) labrum dorsal submarginal arc of setae composed of one plus five simple setae; C) right mandible: canine with 4+4 denticles, prostheca stick-like, apically and distolaterally denticulate; D) left mandible: canine with 3+3 denticles plus a minute intermediate denticle; E) hypopharynx without medial tuft on lingua; F) maxillary palp somewhat longer than galea-lacinia, apically rounded; G) maxilla: distal dentiseta tooth-like, middle and proximal dentisetae trifid, last furcation of proximal seta strongly developed and abducted; H) labial palp segment II slightly produced apicolaterally, segment III conical and pointed; I) fore femur dorsal margin with 19–24 curved, spine-like setae, anterior surface with 4–7 robust setae with median ridge, femoral patch strongly developed; J) fore claw with a row of 12–14 denticles; K) length of gill I 0.4× length of gill IV; L) distal margin of paraproct with 11–14 spines, surface with a patch of notched scales.

#### Description.

**Figure 2.**
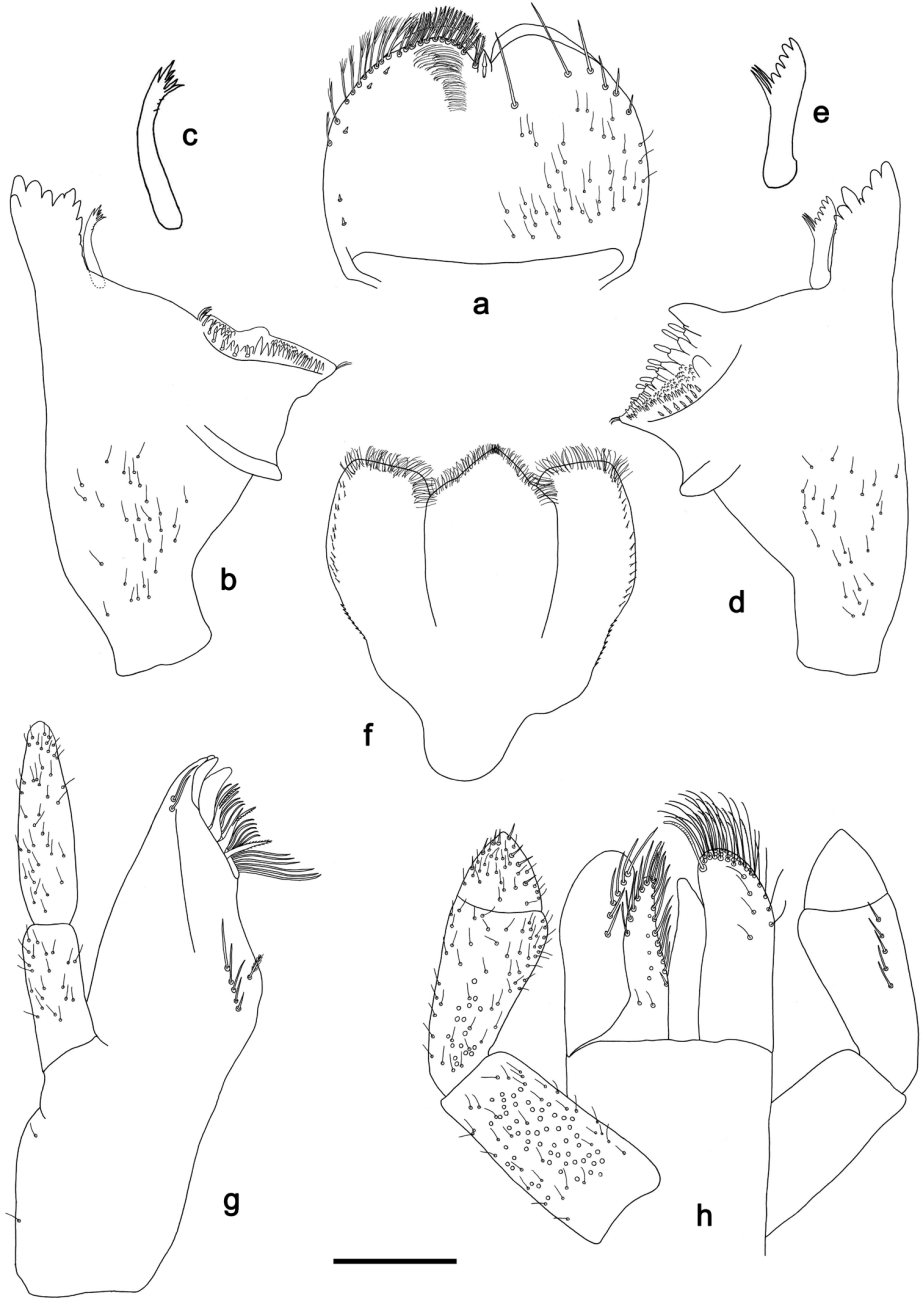
*Tenuibaetisfujitanii* sp. n., larva morphology: **a** Labrum (right: dorsal surface, left: ventral surface) **b** Right mandible **c** Right prostheca **d** Left mandible **e** Left prostheca **f***Hypopharynx***g** Maxilla **h** Labium. Scale bar: 0.1 mm.

**Figure 3.**
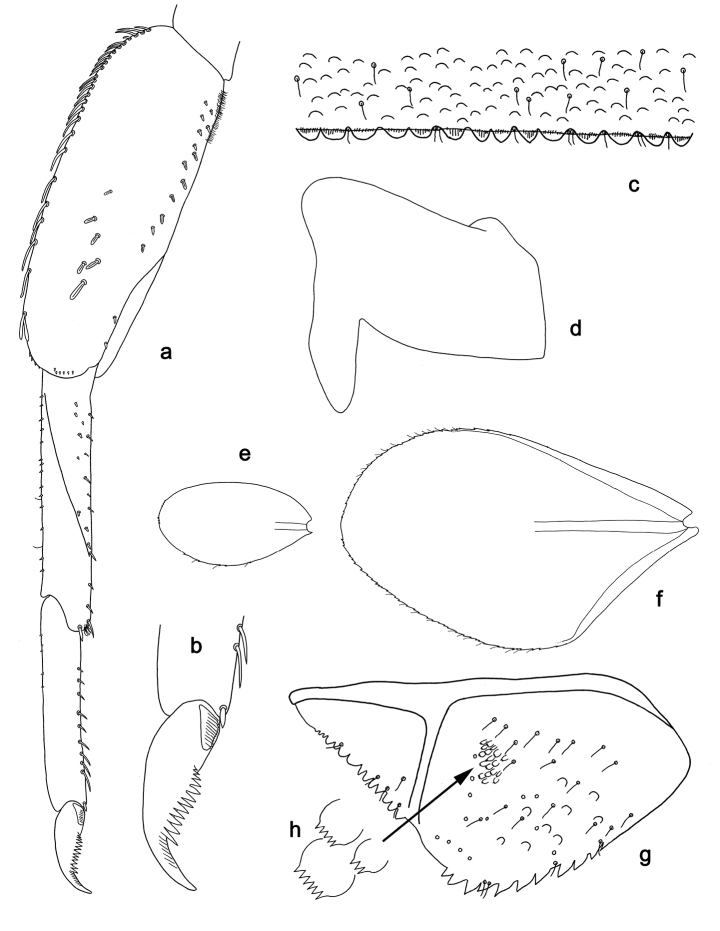
*Tenuibaetisfujitanii* sp. n., larva morphology: **a***Foreleg***b** Fore claw **c***Tergum* IV **d** Left side of metanotum **e** Gill I **f** Gill IV **g***Paraproct*, arrow: notched scales **h** Notched scales on paraproct. **e** and **f** at same scale.

***Larva*** (Figs 1–3). Body length on average 4.5 mm (4.3–4.8 mm); cerci length on average 2.5 mm (2.3–2.8 mm), length of terminal filament on average 1.8 mm (1.7–2.0 mm); antenna: 2.4× as long as head capsule length.

*Colouration.* Head, thorax and abdomen dorsally nearly uniformly brown with slightly darker medial areas as in Fig. 1a, abdominal segment X light brown, head and thorax with bright median, dorsal suture, forewing pads with bright striation. Head with a pair of dark spots at base of clypeus (Fig. 1c). Head, thorax and abdomen ventrally brown, slightly brighter than dorsally. Legs light brown; femur dorsal and apical margin darker brown, distomedial spot on femur darker brown; claws distally dark brown; caudal filaments brown.

*Antenna* with scape and pedicel sub-cylindrical, flagellum with apically rounded spines and fine, simple setae on apex of segments.

*Labrum* (Fig. 2a). Rectangular, length 0.7× maximum width. Medial emargination of distal margin with a small, apically pointed process. Dorsally with many medium, fine, simple setae; submarginal arc of setae composed of one plus five simple setae. Ventrally with marginal row of setae composed of lateral and anterolateral long, feathered setae and medial long, bifid, pectinate setae; ventral surface with five short, spine-like setae near lateral and anterolateral margin.

*Right mandible* (Fig. 2b, c). Incisors fused. Outer and inner set of denticles with four denticles respectively. Prostheca stick-like, apically and distolaterally denticulate (Fig. 2c). Margin between prostheca and mola straight. Tuft of setae at apex of mola present.

*Left mandible* (Fig. 2d, e). Incisors fused. Outer and inner set of denticles with three denticles respectively, plus one minute, intermediate denticle. Prostheca robust, apically with small denticles and comb-shape structure (Fig. 2e). Margin between prostheca and mola straight. Subtriangular process long and slender, above level of area between prostheca and mola. Denticles of mola apically constricted. Tuft of setae at apex of mola present.

Both mandibles with lateral margins almost straight; basal half with fine, simple setae scattered over dorsal surface.

**Hypopharynx** (Fig. 2f). Lingua about as long as superlingua; lingua longer than wide; apically triangular without medial tuft of stout setae; distal half not expanded. Superlingua with distal margin straight; lateral margins rounded; fine, long, simple setae along distal margin; short, pointed setae along lateral margin.

*Maxilla* (Fig. 2g). Galea-lacinia with two simple, robust apical setae under crown. Inner dorsal row of setae with three denti-setae, distal denti-seta tooth-like, middle denti-seta slender, trifid and pectinate, proximal denti-seta slender, trifid and pectinate and with proximal furcation strongly developed and abducted. Medially with one feathered, spine-like seta and 4–5 long, simple setae. Maxillary palp 1.1× as long as length of galea-lacinia; two segmented. Palp segment II 1.6× length of segment I. Fine and simple setae, scattered over surface of segments I and II. Apex of segment II rounded without nipple and without excavation at inner distolateral margin.

*Labium* (Fig. 2h). Glossa basally broad, narrowing toward apex; shorter than paraglossa; inner margin with nine spine-like setae increasing in length distally; apex with two long and one medium, robust, pectinate setae; outer margin with five long, spine-like setae; ventral surface with short, fine, scattered setae. Paraglossa subrectangular, slightly curved inward; apex rounded, with three rows of long, robust, apically pectinate setae; dorsally with 3–4 medium, simple setae; ventrally with an arc of six long, spine-like setae near inner margin. Labial palp with segment I 0.8× length of segments II and III combined. Segment I covered with short, fine, simple setae ventrally and micropores dorsally. Segment II only slightly produced distolaterally; inner and outer margin both with short, fine, simple setae; dorsally with a row of 5–7 long, spine-like, simple setae. Segment III conical, apex slightly pointed; length 0.9× width; ventrally covered with short, spine-like simple setae and short, fine, simple setae.

*Hind wing pads* (Fig. 3d) present, well developed.

**Foreleg** (Fig. 3a, b). Ratio of foreleg segments 1.2:1.0:0.7:0.3. *Femur.* Length ca. 3× maximum width. Dorsal margin with a row of 19–24 long, curved, lanceolate and apically rounded setae; length of setae 0.2× maximum width of femur. Apex rounded; with one pair of clavate setae and two groups of short, stout, pointed setae. Anterior surface with 4–7 robust setae with median ridge. Stout, lanceolate setae along ventral margin; femoral patch strongly developed. **Tibia**. Dorsal margin with a row of short, stout, pointed setae and rare long, fine, simple setae. Ventral margin with a row of short, spine-like setae, on apex one stout, pointed seta and a tuft of fine, simple setae. Anterior surface scattered with stout, lanceolate setae. Tibio-patelar suture present on basal 2/3 area. *Tarsus.* Dorsal margin with a row of short, stout, pointed setae. Ventral margin with a row of curved, spine-like setae. Tarsal claw with one row of 12–14 denticles and with seven stripes apically; claw distally pointed and curved; subapical setae absent.

**Tergum** (Fig. 3c). Surface with scattered U-shaped scale bases and scattered fine, simple setae. Posterior margin of tergum IV with mostly rounded spines, wider than long.

**Gills** (Fig. 3e, f). Present on segments I–VII. Margin with small denticles intercalating fine, simple setae. Tracheae limited to proximal part of main trunk. Gill I as long as ⅔ of length of segment II; gill IV as long as length of segment V and ½ of segment VI combined, 2.3× length of gill I; gill VII as long as length of segment VIII.

**Paraproct** (Fig. 3g, h). Distally not expanded, with 11–13 marginal, stout spines. Surface with U-shaped scale bases, micropores and fine, simple setae, and with a patch of notched scales (Fig. 3h). Posterior extension (cercotractor) with medium, marginal spines.

#### Etymology.

This species is dedicated to Dr Toshihito Fujitani (Japan), who contributed much to the knowledge of the genus *Tenuibaetis*.

#### Distribution.

Java (Indonesia).

#### Type-material.

**Holotype.** Nymph (on slide, GBIFCH00465233), Indonesia, Java, Bogor (downstream the botanical garden), Ciliwung riv, 235 m, 06°35'32"S, 106°48'00"E, 01.05.2010, Jean-Marc Elouard leg. **Paratypes.** 13 nymphs (3 on slides, GBIFCH00465232, GBIFCH00465234, GBIFCH00465235; 10 in alcohol, GBIFCH00515308, GBIFCH00657733, GBIFCH00657755, GBIFCH00657782), same data as holotype. All material deposited in Museum of Zoology Lausanne (MZL).

## Discussion

For the assignment of the new species to *Tenuibaetis* we refer to Kang et al. (1994) and Fujitani et al. (2003a). *Tenuibaetisfujitanii* sp. n. possesses all characters described by these authors: mandibles with smooth margin between prostheca and mola, without setae; labial palpus with segment II poorly expanded at inner distal margin and segment III conical; a well-developed femoral patch; a patch of notched scales on the paraproct; the presence of robust setae with median ridge on the anterior surface of the nymphal femur (Figs 2b, d, h, 3a, g, h).

The new species is clearly distinguished from all other species of *Tenuibaetis* as detailed in Table 1. Notably, it is dorsally brown with slightly darker areas, whereas the other species have more distinct patterns. Additionally, the combination of the following characters differentiates the new species: a relatively short labrum (0.7× shorter than wide), the relatively short maxillary palp (1.1× length of galea-lacinia), the number of setae on the dorsal margin of the femur (19–24) and the shape of the spines at the posterior margin of tergum IV (mostly rounded and wider than long).

**Table 1.** Character states of *Tenuibaetis* species (nymphs).

		*T.fujitanii* sp. n.	* T. flexifemora *	* T. parvipterus *	* T. pseudofrequentus *	* T. arduus *	* T. inornatus *	* T. frequentus *
**Colour**	Dorsal pattern	rather uniform brown	distinct pattern	distinct pattern	distinct pattern	distinct pattern	distinct pattern	distinct pattern
		(Fig. 1a in this study)	(fig. 2 and table 1 in Fujitani et al. 2011)	(fig. 6 and table 1 in Fujitani et al. 2011)	(fig. 9 in Müller-Liebenau 1985, fig. 3 and table 1 in Fujitani et al. 2011)	(fig. 27 in Kang et al. 1994)	(figs 12, 26 in Kang et al. 1994)	(fig. 10 in Müller-Liebenau and Hubbard 1985, fig. 1 in Kubendran et al. 2015)
**Labrum**	Length vs. width	0.7×	0.7×	0.7×	0.8×	0.8×	0.8×	0.7×
	Pattern	absent	absent	absent	absent	absent	U-shaped dark marking	absent
**Maxillary palp**	Length vs. galea-lacinia	1.1×	1.2×	1.2×	1.3×	1.2×	1.15×	1.4×
**Forefemur**	Nb. of dorsal setae	19–24	18–25	14–25	about 14	about 13	?	about 15
**Terga**	Spines at posterior margin	mostly rounded; wider than long	triangular, pointed; longer than wide or about as long as wide	triangular, pointed; wider than long or about as wide as long	triangular, pointed; longer than wide	triangular, blunt; wider than long	triangular, blunt; wider than long	triangular, pointed; longer than wide
***Gills***	Tracheation	basal part of trunk	obscure	obscure	obscure	obscure	distinct, till margins	obscure
	Length gill IV to gill I	2.3×	2.6×	3.6×	2.7×–3.1×	2.3×	1.5×	2.0×
***Paraproct***	Nb. of marginal spines	about 10	10–15	11–15	about 10	about 14	about 11	about 20
**Terminal filam.**	Length vs. cerci	0.7×–0.8×	0.6×–0.7×	0.6×–0.7×	0.5×–0.6×	0.76×	0.65×	0.6×
**Reference**		Present study	Fujitani et al. 2011	Fujitani et al. 2011	Müller-Liebenau 1985	Kang et al. 1994	Kang et al. 1994	Müller-Liebenau and Hubbard 1985
			T. Fujitani, pers. comm.	T. Fujitani, pers. comm.	Kang et al. 1994			Kubendran et al. 2015
					Fujitani et al. 2011			

*Baetisursinus* and *B.hissaricus* were not formally assigned to *Tenuibaetis* so far, but there seems to be no doubt that they belong to this genus. *Tenuibaetisfujitanii* sp. n. is clearly differentiated from *B.ursinus* by the dorsal colour pattern of the larvae (fig. 20 in Kazlauskas 1963, figs 18–29 in Novikova 1991), the shorter maxillary palp (1.1× as long as length of galea-lacinia in *T.fujitanii* sp. n and 1.3× in *B.ursinus*; fig. 24 in Kazlauskas 1963), the dorsal setation of the labrum (fig. 41 in Kluge 1983) and the shape of the spines at the posterior margin of the terga, which are shorter and more rounded in *T.fujitanii* sp. n. (fig. 44 in Kluge 1983, fig. 30 in Novikova 1991). *Tenuibaetisfujitanii* sp. n. is also differentiated from *B.hissaricus* by the dorsal pattern of the larvae (figs 1–16 in Novikova 1991) and the spines at the posterior margin of the terga, which are shorter and more rounded in *T.fujitanii* sp. n. (fig. 17 in Novikova 1991).

We could not obtain any molecular sequences from *T.fujitanii* sp. n. despite several attempts, as the DNA has probably degraded.

Despite sampling nearly 20 localities in Java (and more than 250 sampling localities in the whole of Indonesia), we were unable to find other populations of *Tenuibaetis*. The sampling effort still remains extremely limited regarding the size and diversity of habitats of Indonesia. As *T.fujitanii* sp. n. was collected at a lower altitude and in a highly disturbed habitat, it is likely that the species has a wider distribution, at least in Java. We can also expect that other species of *Tenuibaetis* will be discovered in the future with further samplings in Indonesia and South-East Asia.

## Supplementary Material

XML Treatment for Tenuibaetis fujitanii
